# miRNA-141 attenuates UV-induced oxidative stress via activating Keap1-Nrf2 signaling in human retinal pigment epithelium cells and retinal ganglion cells

**DOI:** 10.18632/oncotarget.14489

**Published:** 2017-01-04

**Authors:** Li-Bo Cheng, Ke-ran Li, Nan Yi, Xiu-miao Li, Feng Wang, Bo Xue, Ying-shun Pan, Jin Yao, Qin Jiang, Zhi-feng Wu

**Affiliations:** ^1^ Department of Ophthalmology, Wuxi Second Hospital, Nanjing Medical University, Wu'xi, China; ^2^ The Affiliated Eye Hospital, Nanjing Medical University, Nanjing, China; ^3^ Department of Hand and Foot Surgery, The Second Affiliated Hospital of Soochow University, Soochow University, Suzhou, China

**Keywords:** miRNA-141, UV, oxidative stress, Keap1, Nrf2 signaling, retinal pigment epithelium cells, retinal ganglion cells

## Abstract

Activation of NF-E2-related factor 2 (Nrf2) signaling could protect cells from ultra violet (UV) radiation. We aim to provoke Nrf2 activation via downregulating its inhibitor Keap1 by microRNA-141 (“miR-141”). In both human retinal pigment epithelium cells (RPEs) and retinal ganglion cells (RGCs), forced-expression of miR-141 downregulated Keap1, causing Nrf2 stabilization, accumulation and nuclear translocation, which led to transcription of multiple antioxidant-responsive element (ARE) genes (HO1, NOQ1 and GCLC). Further, UV-induced reactive oxygen species (ROS) production and cell death were significantly attenuated in miR-141-expressing RPEs and RGCs. On the other hand, depletion of miR-141 via expressing its inhibitor antagomiR-141 led to Keap1 upregulation and Nrf2 degradation, which aggravated UV-induced death of RPEs and RGCs. Significantly, Nrf2 shRNA knockdown almost abolished miR-141-mediated cytoprotection against UV in RPEs. These results demonstrate that miR-141 targets Keap1 to activate Nrf2 signaling, which protects RPEs and RGCs from UV radiation.

## INTRODUCTION

Existing evidences have shown that excessive ultra violet (UV) radiation may induce direct damages to resident retinal pigment epithelium (RPE) cells (RPEs) and retinal ganglion cells (RGCs) [1, 2], which is the major contributor of retinal degeneration [3–5]. Growth evidences have demonstrated that UV radiation in RPEs and RGCs promotes reactive oxygen species (ROS) production, which leads to cell apoptosis [1, 2, 6–10]. Reversely, ROS scavenging could significantly attenuate or even reverse UV damages in RPEs and RGCs [1, 2, 6–10].

NF-E2-related factor 2 (Nrf2) transcriptionally controls the expression of several key anti-oxidative enzymes [11–13]. Expression of these enzymes, *i.e*. heme oxygenase-1 (HO1), NAD(P)H quinone oxidoreductase 1 (NQO1) and γ-glutamyl cysteine ligase catalytic subunit (GCLC) [11], would lead to a profound anti-oxidant response and reduced cytotoxicity [11–13]. Nrf2's activity is mainly controlled by its interaction with Keap1 [11–13]. In the resting conditions, Keap1 association with Nrf2 leads to Nrf2 ubiquitination and degradation [11–13]. Activated Nrf2, for example via phosphorylation at Ser-40, disassociates with Keap1, causing Nrf2 stabilization and accumulation [11–13]. Nrf2 will then translocate to nuclei and binds to antioxidant-responsive element (ARE), dictating transcription of the above enzymes [11–13].

Growth evidences have shown that microRNAs are important in almost all cellular biological processes including oxidative and anti-oxidant responses [14–16]. microRNA deregulation is associated with a number of diseases [14–16]. In the current study, we show that microRNA-141 (“miR-141”) activates Nrf2 signaling via selectively targeting and silencing Keap1, which inhibits UV-induced oxidative stress and apoptosis in RPEs and RGCs.

## RESULTS

### miR-141 expression downregulates Keap1 in human RPEs and RGCs

**Figure 1:**
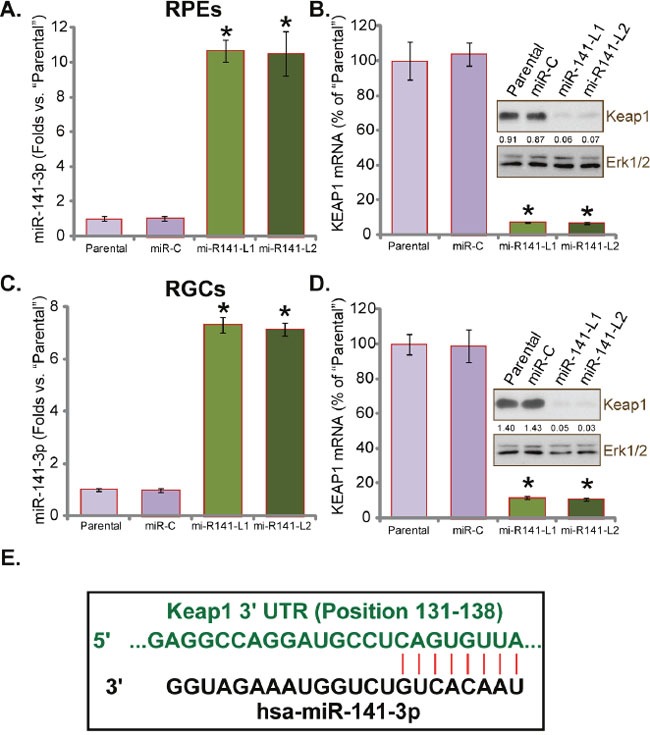
miR-141 expression downregulates Keap1 in human RPEs and RGCs Relative expressions (*vs*. “Parental” cells) of miR-141 and Keap1 (mRNA and protein) in ARPE-19 cells (“RPEs”, same for all figures) **(A)** and **(B)** and primary human RGCs (“RGCs”, same for all figures) **(C)** and **(D)** with miR-141 (two lines, “L1/L2”) or scramble miRNA control (“miR-C”) were shown. **(E)** miR-141-3p selectively targets 3′ UTR of human Keap1 (**E**). Keap1 expression (*vs*. lading Erk1/2) was quantified (**C** and **D**). “Parental” stands for non-transfected parental cells (Same for all figures). For each assay, n=5. Experiments in this figure were repeated four times to insure consistency of results. Data were presented as mean ± standard deviation (SD) (Same for all figures). * *p*<0.05 *vs*. “Parental” cells (**A**–**D**).

In the current study, a miR-141 expression vector was constructed (see Materials and Methods). The miR-141 vector was transfected to ARPE-19 cells (“RPEs”) [6, 17]. Through puromycin selection, two stable ARPE-19 cell lines expressing miR-141 were established, namely “miR-141-L1” and “miR-141-L2”. miR-141 expression in these RPEs was tested. Real-time quantitative PCR (RT-qPCR) assay results in (Figure 1A) showed that miR-141-3p was indeed significantly increased in miR-141-L1/L2 RPEs. Since miR-141-3p could potentially target Keap1 [14, 18, 19], we next analyzed Keap1 expression in these cells. As demonstrated, Keap1 mRNA and protein expressions in miR-141-L1/L2 RPEs were sharply decreased, as compared to control parental RPEs (Figure 1B). The above experiments were also performed in primary human RGCs. Similarly, two stable RGC cell lines expressing miR-141 were established: miR-141-L1/L2 RGCs. These RGCs again expressed high level of miR-141 (Figure 1C), but depleted Keap1 (Figure 1D). These results clearly show that forced-expression of miR-141 downregulated Keap1 in both human RPEs and RGCs. Notably, the non-sense miRNA control (“miR-C”) had no significant effect on expression of miR-141-3p and Keap1 (Figure 1A-1D). Results in (Figure 1E) illustrated that miR-141-3p selectively targets the 3′ UTR of human Keap1 (Position 131-138).

### miR-141 expression stabilizes and activates Nrf2 in human RPEs and RGCs

**Figure 2:**
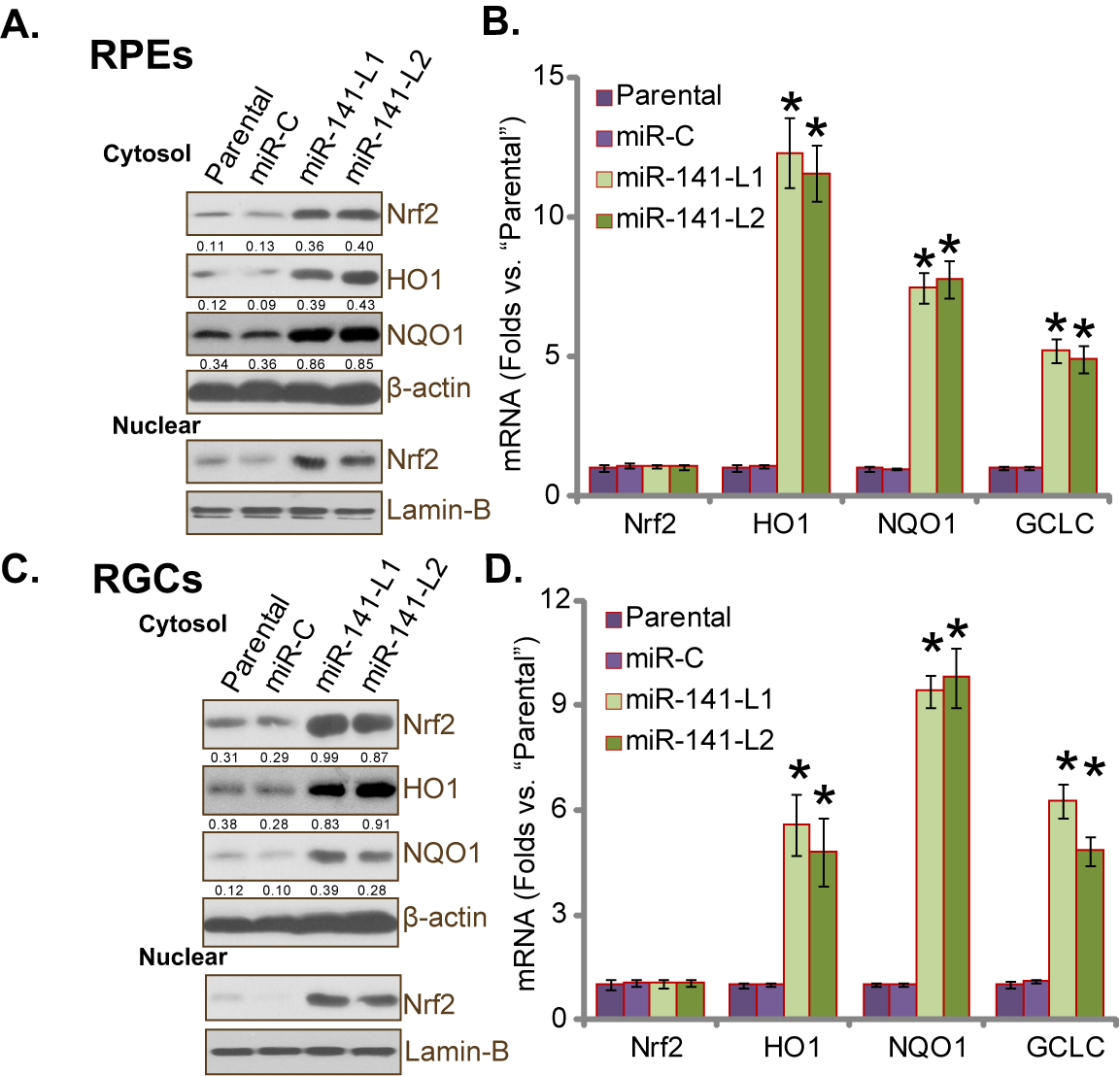
miR-141 expression stabilizes and activates Nrf2 in human RPEs and RGCs Listed protein and mRNA expressions in stable RPEs **(A)** and **(B)** and RGCs **(C)** and **(D)** with miR-141 (two lines, “L1/L2”) or scramble miRNA control (“miR-C”) were examined by Western blot assay (**A** and **C**) and RT-qPCR assay (**B** and **D**), respectively. Nuclear Nrf2 expression was also shown (**A** and **C**, lower panels). Protein expressions of Nrf2, HO1 and NQO1 (*vs*. β-actin) were quantified (**A** and **C**). For each assay, n=5. Experiments in this figure were repeated four times to insure consistency of results. *p<0.05 *vs*. “Parental” cells (**A**–**D**).

Keap1 binds to Nrf2, causing it ubiquitination and proteasome degradation [11]. On the other hand, Keap1 knockdown or loss-of-function mutation should lead to Nrf2 stabilization [11]. Since expression of miR-141 dramatically downregulated Keap1 (Figure 1), its effect on Nrf2 expression was then tested. As demonstrated, forced-expression of miR-141 induced Nrf2 protein accumulation in RPEs (two lines, Figure 2A). Yet, Nrf2 mRNA expression was unchanged after miR-141 expression (Figure 2B). Intriguingly, expression of Nrf2's genes, including *HO1*, *NQO1* and *GCLC* [11], were significantly increased (both protein and mRNA) following miR-141 expression in RPEs (Figure 2A and 2B). We didn't have a decent antibody recognizing GCLC. Remarkably, miR-141 also promoted Nrf2 nuclear translocation in RPEs (Figure 2A). Very similar results were also observed in RGCs, where miR-141 expression stabilized Nrf2 (Figure 2C) and promoted transcription of Nrf2's genes (two RGC lines, Figure 2C and 2D). These results suggest that miR-141 expression induces Nrf2 stabilization and activation in RPEs and RGCs.

### miR-141 expression protects RPEs and RGCs from UV

**Figure 3:**
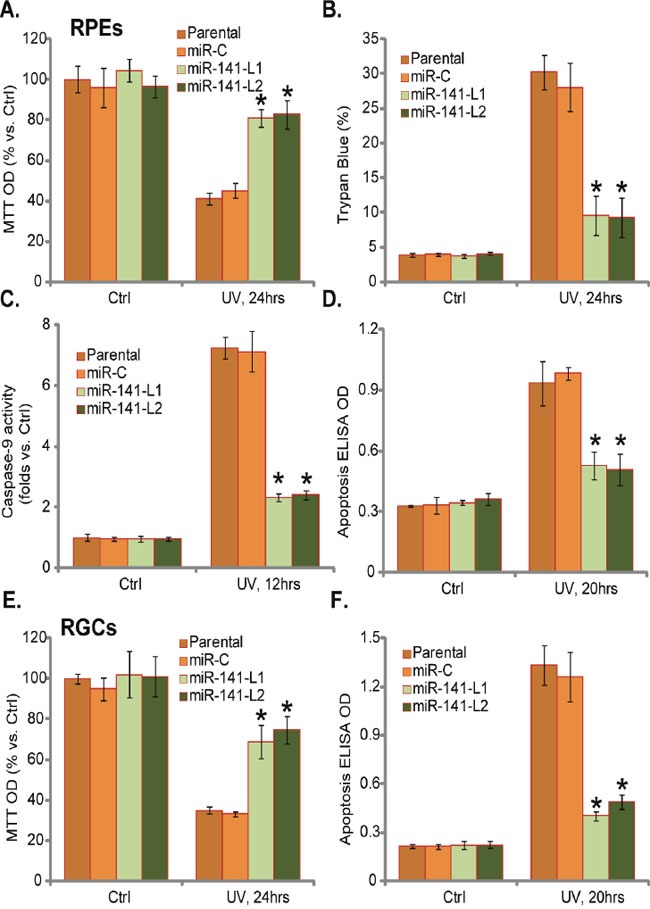
miR-141 expression protects RPEs and RGCs from UV Stable RPEs **(A–D)** or RGCs **(E)** and **(F)** expressing miR-141 (two lines, “L1/L2”) or scramble miRNA control (“miR-C”) were subjected to UV radiation (30 mJ/cm^2^), cells were further cultured for applied time; Cell viability was tested by MTT assay (**A** and **E**); Cell death was tested by trypan blue assay (**B**); Cell apoptosis was tested by the indicated assays (**C**, **D**, and **F**). For each assay, n=5. Experiments in this figure were repeated three times to insure consistency of results. *p<0.05 *vs*. UV-treated “Parental” cells.

Our previous studies have demonstrated that Nrf2 activation could protect RPEs from UV [8, 17, 20]. MTT assay results in (Figure 3A) demonstrated that, as compared to the control cells, the two lines of RPEs with miR-141 over-expression were largely protected from UV. UV-induced a much weaker viability reduction in miR-141-expressing RPEs (Figure 3A). Meanwhile, UV-induced RPE cell death (Figure 3B) and apoptosis (Figure 3C and 3D) were also largely attenuated with miR-141 expression. In line with our previous studies [8, 17, 20], cell apoptosis was tested by caspase-9 activity assay (Figure 3C) and Histone DNA apoptosis ELISA assay (Figure 3D). miR-141-induced cytoprotection against UV was also observed in RGCs, where miR-141 expression dramatically attenuated UV-induced viability reduction (Figure 3E) and apoptosis activation (Figure 3F). Collectively, miR-141 expression significantly protects RPEs and RGC from UV.

### miR-141 expression inhibits UV-induced ROS production in RPEs and RGCs

**Figure 4:**
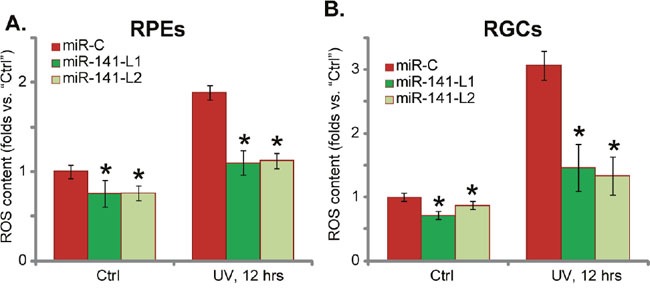
miR-141 expression inhibits UV-induced ROS production in RPEs and RGCs Stable RPEs **(A)** and RGCs **(B)** expressing miR-141 (two lines, “L1/L2”) or scramble miRNA control (“miR-C”), were subjected to UV radiation (30 mJ/cm^2^), cells were further cultured for 12 hours, ROS production was tested by the carboxy-H2DCFDA FACS assay, and its level was normalized to the untreated control (“Ctrl”). For each assay, n=5. Experiments in this figure were repeated three times to insure consistency of results. *p<0.05 *vs*. “miR-C” cells.

Nrf2 is a well-established anti-oxidant signaling [11, 21]. Above results demonstrated that miR-141 activated Nrf2 signaling in RPEs and RGCs, next we analyzed its effect on UV-induced ROS production. As demonstrated, the intracellular ROS content was significantly increased following UV radiation in both RPEs (Figure 4A) and RGCs (Figure 4B). Remarkably, forced-expression of miR-141 significantly attenuated ROS production in UV-treated RPEs (Figure 4A) and RGCs (Figure 4B). The ROS content in miR-141-expressing cells following UV radiation was comparable to the untreated control level (Figure 4A and 4B), indicating the potent anti-oxidant activity by miR-141 expression. Notably, the basal ROS content was also lower with miR-141 expression in both RPEs and RGCs (Figure 4A and 4B). Together, miR-141 expression largely inhibits UV-induced ROS production in RPEs and RGCs.

### antagomiR-141 downregulates Nrf2 and sensitizes UV damages in RPEs

**Figure 5:**
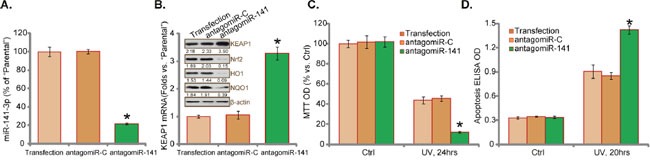
antagomiR-141 downregulates Nrf2 and sensitizes UV damages in RPEs Expression of miRNA-141 **(A)** Keap1 mRNA **(B)** and listed proteins (**B**) in RPEs transfected with antagomiR-141 or non-sense control antagomiR (“antagomiR-C”) were shown. Cells were also subjected to UV radiation (30 mJ/cm^2^) and cultured for applied time, and cell viability was tested by MTT assay **(C)** Cell apoptosis was tested by Histone DNA ELISA assay **(D)**. “Transfection” indicates transfection reagents only. Protein expressions of Keap1, Nrf2, HO1 and NQO1 (*vs*. β-actin) were quantified (**A** and **C**). For each assay, n=5. Experiments in this figure were repeated three times to insure consistency of results. *p<0.05 *vs*. “antagomiR-C” cells.

Next, antagomiR-141, an anti-sense RNA molecule complementary to miR-141 [22], was transfected to RPEs. RT-qPCR assay results in (Figure 5A) confirmed that antagomiR-141 transfection indeed downregulated miR-141 in the RPEs. Consequently, Keap1 mRNA and protein expression was upregulated (Figure 5B). On the other hand, Nrf2 and HO1/NOQ1 expressions were dramatically reduced (Figure 5B). Remarkably, RPEs with antagomiR-141 were more vulnerable to UV damages (Figure 5C and 5D). UV radiation induced profound cell death (Figure 5C) and apoptosis (Figure 5D) in the RPEs with antagomiR-141. Transfection of the non-sense control antagomiR (“antagomiR-C”) didn't change Keap1-Nrf2 signaling nor UV-induced cell death (Figure 5A-5D). These results imply that miR-141 depletion by expressing antagomiR-141 downregulates Nrf2 and exacerbates UV damages in RPEs.

### Nrf2 knockdown abolishes miR-141-meidated RPE cytoprotection against UV

**Figure 6:**
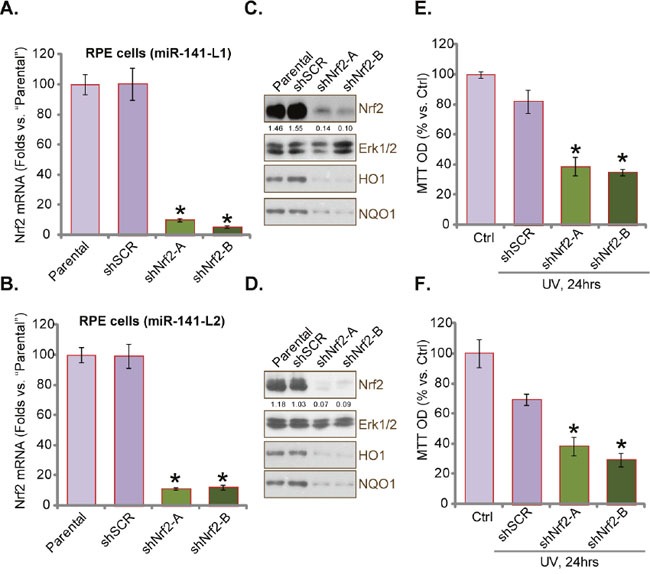
Nrf2 knockdown abolishes miR-141-meidated RPE cytoprotection against UV miRNA-141-expressing RPEs (“L1/2”) were infected with lentiviral Nrf2 shRNA (“shNrf2-A/-B”, with non-overlapping sequence) or scramble control shRNA (“shSCR”), expression of Nrf2 mRNA **(A)** and **(B)** and listed proteins **(C)** and **(D)** were shown. Above cells were also irradiated with UV (30 mJ/cm^2^) and cultured 24 hours, and cell viability was tested by MTT assay **(E)** and **(F)**. Nrf2 expression (*vs*. loading Erk1/2) was quantified (**C** and **D**). For each assay, n=5. Experiments in this figure were repeated three times to insure consistency of results. *p<0.05 *vs*. “shSCR” cells.

The results above have shown that miR-141 activates Nrf2 signaling and protects cells from UV. Next, we wanted to explore the link between the two. shRNA strategy was utilized to stably knockdown Nrf2 in miR-141-expressing RPEs. Two different Nrf2 shRNAs (“shNrf2-A/-B”, see Materials and Methods) with non-overlapping sequences were applied, each of them efficiently downregulated Nrf2 expression (protein and mRNA) in miR-141-expressing RPEs (“L1/2”) (Figure 6A-6D). Meanwhile, HO1 and NOQ1 expressions were also downregulated in the above miR-141-expressing RPEs (Figure 6C and 6D). Consequently, when Nrf2 was depleted, these miR-141-expressing RPEs became vulnerable or re-sensitive to UV (Figure 6E and 6F). In another words, Nrf2 depletion almost abolished miR-141-mediated cytoprotection against UV (Figure 6E and 6F). Thus, Nrf2 stabilization and activation should be the main reason of miR-141-induced anti-UV actions in RPEs.

## DISCUSSION

Nrf2-inducing agents were shown to result in decreased oxidative stress and significant cytoprotection [19]. For example, our previous study has shown that SC79, a novel Akt activator [17, 23], protects RPEs from UV via activating Nrf2 signaling [17]. Further, Zhang *et al*., demonstrated that Salvianolic acid A-induced RPE cell protection against oxidative stress requires Nrf2-HO1 signaling [20]. Similarly, Li *et al*., showed that Nrf2 activation by 3H-1,2-dithiole-3-thione (D3T) offers dramatic cytoprotection in UV-radiated RPEs [8].

In this study, we showed that miR-141 expression activated Nrf2 signaling in both RPEs and RGCs. First, miR-141 targeted and degradated Nrf2 inhibitor Keap1 in the above eye cells. Second, Nrf2 was stabilized in the miR-141-expressing cells, which was evidenced by upregulation of Nrf2 protein, but not mRNA. Third, stabilized Nrf2 was shown to translocate to cell nuclei, leading to transcription of several ARE-genes (*HO1*, *NOQ1* and *GCLC*). Importantly, activation of Nrf2 by miR-141 expression significantly ameliorated UV-induced oxidative stress and subsequent death/apoptosis of RPEs and RGCs. shRNA-mediated knockdown of Nrf2 in these cells, on the other hand, almost abolished miR-141-cytoproteciton against UV. We therefore conclude that miR-141 protects RPEs and RGCs from UV radiation via activating Nrf2-Keap1 signaling.

To our best knowledge, this is the first report showing potential biological functions of miR-141 in eye cells. There are at least twenty-seven potential target genes of miR-141 have been characterized thus far, which were predicted by the software including PicTar, TargetScanS, and miRanda [24]. One of these targets is Keap1 [18, 19]. Our results showing miR-141 activates Nrf2 signaling via targeting Keap1 in RPEs and RGCs were consistent with other studies [18, 19]. For example, van Jaarsveld *et al*., showed that miR-141 depletes Keap1 to decrease cisplatin sensitivity in ovarian cancer cells [18]. Similarly, miR-141 activates Nrf2-dependent antioxidant pathway via silencing keap1 to confer resistance to 5-FU [19].

UV radiation and oxidative stress induce significant damages to RPEs and RGCs. These are the underlying pathological mechanism of AMD and other retinal degenerative diseases [3–5]. The results of the current study showing that miR-141 potently activates Nrf2 signaling, which attenuates UV-induced oxidative stress and RPEs/RGCs damages.

## MATERIALS AND METHODS

### Reagents, chemicals and antibodies

The anti-β-actin antibody was obtained from Sigma (A1978, 42kD, St. Louis, MO). Other antibodies utilized in this study were purchased from Cell Signaling Tech (Shanghai, China): HO-1 (#70081, 28kD), NQO1 (#3187, 29kD), Nrf2 (#12721, 98kD), KEAP1 (#8047, 60kD), Erk1/2 (9102, 42/44kD) and Lamin B1 (#13435, 55kD). Cell culture reagents were obtained Gibco (Suzhou, China).

### Culture of human RPEs

Culture of human retinal pigment epithelial cells (ARPE-19 cell line) was described previously [8, 25, 26].

### Primary culture of human RGCs

Fresh donor human eyes were obtained from ocular trauma patients administrated at the authors institutions. The human retina was separated very carefully from the globe and dissected to give a flat retinal preparation, which was then maintained in the DMEM/HamF12 medium plus 50 μg/mL gentamicin in the 35mm culture dish (Corning, Suzhou, China). Retinal cells were then isolated by enzymatic dissociation [27], and were cultured in F12 medium with 12% serum, plus nerve growth factor (NGF, Sigma) and basic fibroblast growth factor (bFGF, Sigma). RGCs were positive for neurofilaments and Thy-1, but were negative for glial fibrillary acidic protein (GFAP) [27]. Primary RGCs at passage 3-7 were utilized for experiments. Written-informed consent was obtained from each patient. Experiments and the protocols requiring clinical samples were approved by the Ethics Review Board (ERB) of all authors’ institutions. All investigations were conducted according to the principles expressed in the Declaration of Helsinki as well as national/international regulations.

### Forced miR-141 expression

The pre-has-miR-141 was obtained from Applied Biosystem (Shanghai, China), which was sub-cloned into pSuper-puro-GFP vector (OligoEngine, Seattle, WA) to construct miR-141 expression vector. The miR-141 construct or the empty vector was transfected to the RPEs and RGCs through Lipofectamine 2000 reagents (Invitrogen, Shanghai, China). The transfection took 48 hours. Afterwards, puromycin (1.0 μg/mL) was added to the complete medium to select stable cells, which lasted 3-4 passages, until at least 95% of cells were GFP positive. Control cells were transfected with non-sense scramble microRNA-control (“miR-C”) (Applied Biosystem). Mature has-miR-141-3p expression in the stable cells was tested by RT-qPCR assay.

### antagomiR-141 expression

RGCs or RPEs were transfected with 200 pmol of miR-141 inhibitor (“antagomiR-141”, synthesized by GenePharm, Shanghai, China) [22] or negative control antagomiR (“antagomiR-C”, GenePharm) via the Lipofectamine2000 (Invitrogen) reagents. After 48 hours, miR-141 expression was validated by RT-qPCR assay.

### Real-time quantitative PCR analysis

After applied treatment, cellular RNA was extracted via the Trizol reagents (Invitrogen), which was then utilized to perform the reverse transcription assay [17, 20]. The PCR reaction mixture contained 1× SYBR Master Mix (Applied Biosystem), 500 ng RNA together with indicated primers. Real-Time quantitative PCR (“RT-qPCR”) was performed by the ABI Prism 7300 Fast system (Shanghai, China). The ^ΔΔ^Ct method was utilized to quantify mRNA expression, and GAPDH was tested as an internal control. Primers were described in our previous studies [8, 20] and in published literatures [28]. Expression of mature hsa-miR-141-3p was examined by the same ABI Prism 7300 Fast system. Primers for hsa-miR-141-3p were described previously [29]. All the primers were synthesized by Genepharm (Shanghai, China).

### UV radiation

UV radiation (UVB and UVA2) to cultured cells was performed as reported [6–8, 17].

### Cell viability and cell death assays

Following treatment, the survival of cells was tested via the routine MTT assay; Cell death was examined by trypan blue staining assay. The detailed protocols were described in our previous publications [17, 20, 30].

### Cell apoptosis assays

After treatment, cell apoptosis was examined by previously described assays, including Histone DNA apoptosis enzyme-linked immunosorbent assay (ELISA) assay and the caspase-9 activity assay. Detailed protocols were described in our previous studies [8, 17, 20, 31–33].

### ROS assay

As previously reported [20, 34], the intracellular ROS production was measured by the carboxy-H2DCFDA (Invitrogen, Shanghai, China) assay. After applied treatment, cells were stained with 1 μM of carboxy-H2-DCFDA at 37°C for 30 min, which were then tested via flow cytometry (BD bioscience). Relative H2DCFDA intensity (vs. untreated control cells) was recorded to reflect cellular ROS intensity [20, 34].

### Western blot assay

Western blot assay was performed as described [7, 20, 31]. Each band was quantified (in total gray) by ImageJ software, and was normalized to the indicated loading control [7]. Each lane was loaded with exact same amount of protein lysates (30 μg per sample). Same set of lysate samples were run in sister gels to test different proteins. For detection of nuclear proteins, cell nuclei were isolated by the nuclei isolation kit from Sigma [20].

### Nrf2 shRNA knockdown

The two non-overlapping lentiviral Nrf2 shRNAs, Nrf2 shRNA-A (sc-37030-V) and Nrf2 shRNA-B (sc-44332-V), along with lentiviral scramble control shRNA, were purchased from Santa Cruz Biotech (Santa Cruz); The lentiviral shRNA (10 μL/mL) was added directly to cultured cells for 36 hours. Cells were then subjected to puromycin (1.0 μg/mL) selection for another 6 days. Nrf2 expression in the stable cells was tested by Western blot and RT-qPCR assays.

### Statistical analysis

Quantitative results were normalized to the control values of each assay, and were presented as mean ± standard deviation (SD). Data were analyzed by one-way ANOVA. Significance was chosen as *p* < 0.05.
